# Impacts of crude oil market on global economy: Evidence from the Ukraine-Russia conflict via fuzzy models

**DOI:** 10.1016/j.heliyon.2023.e23874

**Published:** 2023-12-18

**Authors:** Muhammad Bilal, Muhammad Aamir, Saleem Abdullah, Faisal Khan

**Affiliations:** a Department of Statistics, Abdul Wali Khan University, Mardan, Pakistan; b Department of Mathematical Sciences, Balochistan University of Information Technology, Engineering and Management Sciences (BUITEMS), Quetta, Pakistan; c Department of Mathematics, Abdul Wali Khan University, Mardan, Pakistan; d Department of Electrical and Electronic Engineering, College of Science and Engineering, National University of Ireland Galway, Ireland

**Keywords:** Autoregressive integrated moving average, Fuzzy time series, Optimization, Forecasting and MAPE

## Abstract

The increasing Russia-Ukraine crisis is without a doubt Europe's most prominent conflict since World War II, changing the dynamics of the oil and other key markets. Because the oil market has traditionally interacted with other financial and commodity markets, it will be intriguing to examine how it interacts with substantial financial assets amid market volatility induced by a conflict. The goal of this study is to propose a fuzzy time series (FTS) model and to compare its competitiveness to existing fuzzy time series (FTS) models, Autoregressive Integrated Moving Average (ARIMA) model and some machine learning methods i.e. Artificial Neural Networks (ANN), Support Vector Machine (SVM) and XGBoost models. We considered changes in the partitioning universe of discourse, optimization of parameters method(s), and interval estimation to make the forecast accuracy more precise forecasting than traditional methods via MAPE. The event-based data results show the proposed fuzzy time series model is outperforming all the competitive methods in the study. Furthermore, the proposed model forecasting shows a future decline tendency in WTi market crude oil prices (US$/BBL) after being at the record highest level, which is good news for the worldwide economy.

## 1 Introduction

Crude oil is one of the most challenging markets to forecast because it is influenced by so many conflicting crosscurrents, such as supply and demand, global economic health, geopolitics, and the global monetary and regulatory environment. When there is a war in an oil-producing country, oil prices rise. It's one of the most foreseen market developments. In the short term, two major geopolitical developments could have an immediate and negative impact on the oil price: the first is an escalation of the Russia-Ukraine conflict, which could disrupt Russian oil and gas supplies to the European Union; and the second is a worsening of the situation in Iraq, which could affect its oil infrastructure and production (EU). In the long run, however, the major risk to global oil supply and the price of oil comes from the Arab Gulf nations' rapidly expanding domestic oil demand and lack of economic diversification. According to the research [1], an interruption in Iraq's oil output could boost oil prices to more than $140/barrel, while a disruption in Russian oil supply to the EU could easily add $20-$30 to the price of oil. It will also assert that unless the Arab Gulf countries drastically cut their domestic oil use or replace oil with nuclear and solar energy in power generation and water desalination, their oil exports would almost likely fail by 2032. The conflict has had a substantial influence on the global economy, causing growth to stagnate and expenses to rise. Aside from the pain and humanitarian issues brought about by Russia's incursion of Ukraine, the global economy will be affected from slower growth and higher pricing. The consequences will be felt through three major channels. One, higher commodity costs, such as food and energy, will push up inflation further, reducing buying power and influencing demand. Two economies, particularly those next to one other, will see disruptions in commerce, logistics processes, and remittances, as well as an unprecedented rise in refugee flow. Third, falling company confidence and rising investor concern would exert downward pressure on asset prices, tightening economic circumstances and perhaps promoting capital outflows from developing nations. Russia and Ukraine both have substantial resources. Russia and Ukraine are major resource producers, and supply disruptions have driven up worldwide prices, notably for oil and natural gas. Wheat has reached a record high, accounting for 30 % of Ukraine and Russia's global exports. Nations with direct trade, tourism, or financial exposure will suffer additional pressures in addition to global spillovers. Oil-importing nations may run greater budget and trade deficits, as well as face stronger inflationary pressures; nevertheless, certain exporters, particularly those in the Middle East and Africa, may benefit from higher prices. Higher food and fuel costs are expected to exacerbate the risk of instability in a number of nations, ranging from Sub-Saharan Africa and Latin America to the Caucasus and Central Asia, while food insecurity in Africa and the Middle East is expected to intensify. These repercussions are difficult to foresee, but we believe our growth forecasts will be revised downward next month when we provide a more comprehensive picture of our World Economic Outlook and regional reviews. Long-term, the conflict may fundamentally alter the global economic and geopolitical order as oil trade moves, supply lines reorganize, payment networks split, and governments review their reserve currency holdings. The danger of economic fragmentation, particularly in commerce and technology, grows as geopolitical conflict escalates.

In the intricate web of global economics, few events carry as profound an impact as geopolitical conflicts, and the ongoing Ukraine-Russia war stands as a testament to this reality. As the world watches with bated breath, the tendrils of this conflict extend far beyond national borders, intertwining with the complex tapestry of the WTI crude oil market and reverberating throughout the global economy. Crude oil, that lifeblood of modern industry, is uniquely sensitive to geopolitical tremors. Supply and demand dance a precarious duet, influenced not only by economic health but also by the unpredictable choreography of political forces. In this intricate ballet, a war in an oil-producing region serves as a crescendo, sending oil prices soaring in anticipation of disruption. Thus, it is no surprise that market experts are closely monitoring the Ukraine-Russia conflict, identifying key triggers that could resonate with global oil prices. In the immediate horizon, the conflict's escalation holds the potential for swift and unfavorable consequences. The specter of a Russia-Ukraine confrontation looms large, casting shadows over Russian oil and gas supplies to the European Union. Simultaneously, the fragility of Iraq's oil infrastructure underscores the delicate balance between geopolitical instability and oil production. The ripple effects of these potential disruptions are felt not merely as localized tremors, but as seismic shifts in the global oil market landscape. Peering into the distant horizon, the landscape becomes equally intriguing. Beyond the immediate price hikes lies a more formidable challenge – the burgeoning oil demand of Arab Gulf nations. With domestic needs surging and economic diversification lagging, the global oil supply chain faces a reckoning. Research suggests that even an interruption in Iraq's oil output could jolt prices beyond $140 per barrel, while disruptions in Russian oil supplies could inject an additional $20-$30 premium. Meanwhile, the looming threat of dwindling Arab Gulf oil exports by 2032 underscores the urgency of energy diversification. But the impact of this conflict doesn't stay confined to the world of commodities and markets. Its tendrils wind through the corridors of economies and societies alike, triggering a symphony of repercussions. As growth stalls and costs rise, the global economy feels the tremors of uncertainty. A multi-faceted disruption unfolds: commodity costs surge, weighing down purchasing power and dampening demand. Trade routes falter, cascading into disruptions in logistics, commerce, and remittances, and swelling the ranks of displaced populations.

The psychological toll is equally undeniable. Plummeting business confidence and investor trepidation conspire to tighten the economic vise. Asset prices falter under the weight of uncertainty, potentially triggering capital outflows from emerging economies. The intertwined destinies of Russia and Ukraine, both resource-rich nations, come into stark focus. Disruptions in supply chains send shockwaves across global prices, leaving no corner of the world untouched. From the fertile plains of wheat to the intricate webs of trade, the impact is far-reaching, fostering a heightened sense of interdependence in a world already grappling with complexities. As economists scrutinize their forecasts and policymakers strategize their moves, a common thread emerges: uncertainty. Predicting the full extent of the impact remains an elusive endeavor, as geopolitical intricacies continue to intertwine with economic realities. Yet, one thing is certain – the echoes of the Ukraine-Russia conflict will continue to resonate through global oil markets and reverberate across economies, weaving an intricate narrative of interconnectivity in an ever-evolving world. The past studies relevant to the study is briefly discussed in the literature review section.

## 2 Literature review

The current and future research [2] explores the effects of economic shocks on areas such as Europe, the Caucasus, Central Asia, the Middle East, North Africa, Sub-Saharan Africa, the Western Hemisphere, Asia and the Pacific, and Global Shocks. Researchers [3,4] identified the ongoing Russia-Ukraine conflict as the most significant war in Europe since World War II, affecting the dynamics of oil and other important markets and negatively impacting the global economy as a whole. Their research was further enlightened by Ref. [5], who emphasized that the Eurozone manufacturing purchasing managers' index (PMI) fell in the month of the invasion and concluded as food and petroleum prices rose rapidly due to irreparable infrastructure damage. They express their sympathy to Ukraine, which suffered a more severe impact from the invasion than Russia and the entire Eurozone, and they extend it to the world economy. Researchers [6] examined the global GDP further and attempted to present 2023 GDP forecasts for various nations, which would have a detrimental impact on their economies.

The researchers [7], used an event research technique to assess the influence on stock markets across nations following the Russian invasion on February 24, 2022. They observed that EU nations have seen a large drop in cumulative anomalous returns, although enterprises in other countries outside of the war appear to be unaffected. They also believe that economic considerations will be the most compelling argument for Russian administrations to end their conflict in Ukraine. Their research was bolstered by Ref. [8], who urged international leaders to persuade Russia to leave Ukraine or face punitive sanctions.

The authors [9] investigated the transmission of returns and volatility in the commodities universe in the aftermath of the Ukraine war. The overall volatility spillover rises from 35 % to 85 %, surpassing the amount observed during the epidemic. Crude oil becomes a net transmitter of return spillovers, while wheat and soybeans become net receivers. Silver, gold, copper, platinum, aluminum, and sugar become net volatility transmitters. Geopolitical threat spillover indices are caused by Granger. High levels of return and volatility have been linked to high levels of geopolitical risk. Their research on geopolitical risk was expanded upon by Ref. [10], which investigated the impact of geopolitical risks caused by the Russian-Ukrainian conflict on Russia, European financial markets, and global commodity markets using time- and frequency-based time-varying parameter vector autoregression (TVP-VAR) approaches. They investigated the geopolitical aspects and repercussions of gas supply and demand in Europe during the ongoing war [11].

As an event case study [12], examined the impact of a Russian invasion of Ukraine in 2022, concluding that the invasion created negative cumulative abnormal returns for global stock market indices, albeit with diverse consequences. Economic globalization, as measured by GDP-scaled trade, is inversely related to event-day and post-event returns, according to a cross-sectional study. Markets in NATO nations showed better returns, consistent with the projected economic boost of military preparation. Their research is further mirrored in Ref. [13], where equities more vulnerable to the regulatory concerns of the transition to a low-carbon economy did better, implying that investors expect the change to decelerate. Analysts have also raised their profit expectations for these stocks. The stock price consequences for transition risk were especially substantial in the United States. The impacts on transition risk were less strong or perhaps inverse in Europe. Stocks with low-carbon transition potential gained, presumably because market players anticipate stronger governmental responses supporting renewable energy sources in the face of Europe's considerable reliance on Russian oil and gas. As a result, investors anticipate that the pace of transition to a low-carbon economy will differ between the United States and Europe. The analysis accounts for a variety of Environmental, Social, and Governance (ESG) indicators. Companies that mentioned inflation more frequently in analyst conference calls did badly. Internationally focused corporations performed poorly, and investors were particularly concerned about companies' exposure to China.

The researchers [13], investigated significant environmental contamination as well as several other livelihood concerns. They think that the Russian-Ukrainian conflict created a tsunami that had a significant influence on the global economy, geopolitics, and food security. Because of the grave humanitarian crisis, the environmental consequences have been disregarded. However, because of the fierce warfare, the consequences will be tremendous, resulting in an environmental disaster. The violence is already influencing places outside of Ukraine (explosions in Russia and Moldova territory). The prolonged battle causes significant air pollution and greenhouse gas emissions. In addition, combat actions were carried out near the Zaporizhzhia nuclear power station (the largest in Europe) and Chornobyl, heightening fears of radioactive leakage. Deforestation and habitat degradation are wreaking havoc on biodiversity, with serious consequences for species. Bombing, trenching, and tunnel excavations will almost certainly have a detrimental influence on soil deterioration and landscape morphology. This is especially important because Ukraine has some of the most fertile soils in the world (Chernozem), which affects food production. Water availability and quality are anticipated to suffer as a result of infrastructure destruction and pollution movement to water reservoirs. The ecosystem services provided will most likely be severely harmed because deforestation reduces ecosystems' ability to manage air pollution or temperature. Soil pollution will impede food production, and the erosion of landscape aesthetics, cultural assets, and social cohesiveness will have a significant impact on cultural services. Finally, the consequences for human health are already enormous. However, it can be significantly greater due to high levels of pollution and deterioration of sanitary conditions. The consequences of this conflict are quite unknown.

The key contributions of this study are as under.

• Proposed Fuzzy Time Series Model.
• Considerable changing in UoD.
• Forecasting through interval estimation.
• Relative Efficiency.

## 3 Research methodology

In this research study, we have proposed a new FTS model in comparison to FTS Stevenson and Porter Model referred to as the SP model [14] and Autoregressive Integrated Moving Average (ARIMA). By using the percentage change of the dataset for forecasting as UoD, on which fuzzy sets are given, the interval of variations of such indicators as the year-to-year percentage change rates, called “chain growth rates”.

Consider a time series of an economic indicator, denoted by $y_{i}$, i=2,3,…,n. For modeling this time series, the considered method uses the following indicator as chain growth rate in eq. (1):

(1) $$T_{i}=\left(\frac{y_{i}}{y_{i‐1}}‐1\right)\times 100\%$$

Hereafter, the model contains the following steps.

Step 1 Determine the UoD as the Set U, where $U=\left[\min\;T_{i},\max\;T_{i}\right]$, i=1,2,3,…,n and divide into equal “m” intervals, obtained by eq. (2) as:

(2) m=1+3.3logN

Where “m” is the class interval and “N” is the total number of observations in “U”.

Step 2 After frequency distribution, naturally, large classes are divided into smaller partitions for accuracy. In our dataset, we have divided the larger three classes into 5-3-11 sub-intervals respectively for improving accuracy.

Step 3 Fuzzy sets $X_{k}$, k=1,2,3,…,m on each partition interval as the triangular fuzzy number, which carriers are defined by three values: lower limit, midpoint, and upper limit. For the actual dataset, it is to determine which fuzzy set will describe each value. So that fuzzifies the dataset into initial series.

Step 4 Defuzzification of the fuzzy set to crisp values is done by eq. (3):

(3) $$t_{j}=\left\{ \begin{array}{cc} \frac{1+0.5}{\frac{1}{a_{1}}+\frac{0.5}{a_{2}}} & j=1\\ \frac{0.5+1+0.5}{\frac{0.5}{a_{j-1}}+\frac{1}{a_{j}}+\frac{0.5}{a_{j+1}}} & 2\leq j\leq m-1\\ \frac{0.5+1}{\frac{0.5}{a_{j-1}}+\frac{1}{a_{j}}} & j=m \end{array}\right.$$

Where $a_{j-1}$, $a_{j}$, and $a_{j+1}$ are the midpoints of the interval of fuzzy sets $X_{k-1}$, $X_{k}$, and $X_{k+1}$ carriers respectively.

Step 5 Determine the predicted values for each level of our proposed model as under in eq. (4):

(4) $${\overset{\wedge }{y}}_{i}=y_{i-1}+\frac{t_{j}}{100},i=2,3,4,...,n$$

As in Ref. [14], the forecasting results by eq. (4) are supposed to have a better accuracy rate than other FTS models as well as conventional time series models. However, the accuracy may be further improved if the $t_{j}$ parameters rather than specified values as in eq. (3) are estimated and the optimized values are used for forecasting the future value. In this study, we have implemented and will be discussed later.

Case Study: Crude Oil Prices ($/BBL) during Russia Ukraine War.

**Fig. 1:**
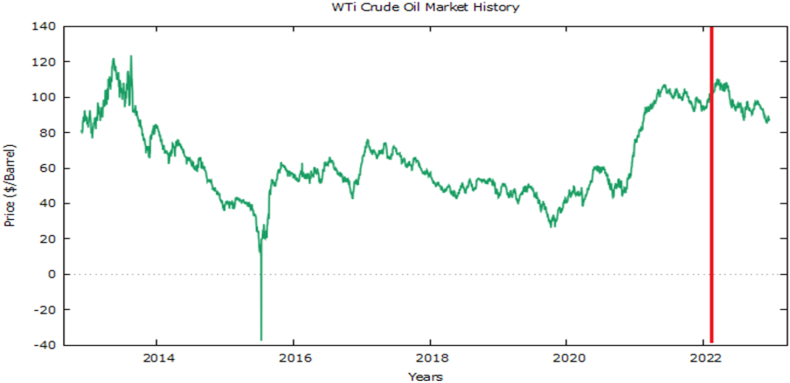
History of WTi market.

In Fig. 1, international market WTi crude oil daily prices ($/BBL) 10 years data has been presented i.e. from Nov 26, 2012, to Nov 23, 2022. Before and after Ukraine- Russia War by putting a dissection line on February 17, 2022, to onward as war data which is our case study for this article. During COVID-19, prices recorded a historic low while consecutively high for crude oil international since the war break out, therefore its assessment and forecast are our interest area to investigate.

**Fig. 2:**
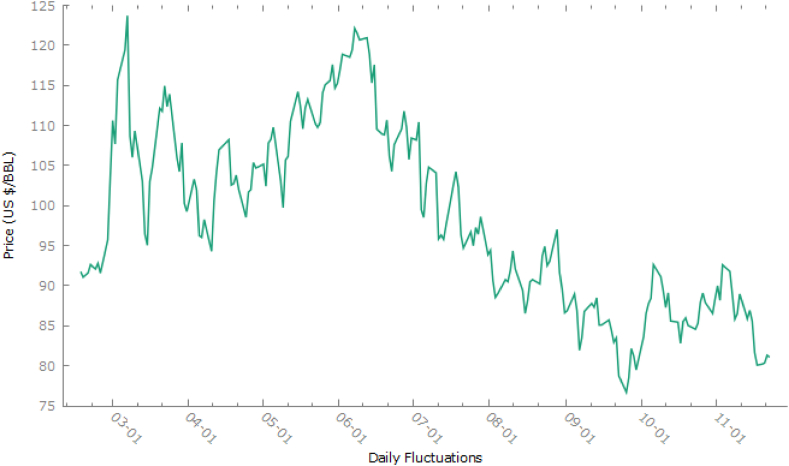
WTi market daily crude oil prices ($/BBL) during Ukraine- Russia war.

In Fig. 2, WTi market crude oil prices ($/BBL) during Ukraine-Russia war daily data has been presented for a clearer insight into the case data. During the war, we see that minimum crude oil prices ($/BBL) were recorded as 85.56 and maximum as 110.53. By eq. (1), we get the growth rate (T) as under.

In Table 1, Growth Rate is displayed against corresponding values, we observed that it is varying from −0.12126 to 0.083544 as the minimum and maximum values. We form a universe of discourse as U=[−0.12126,0.083544] and its frequency distribution using class boundaries limits system by using eq. (2), huge contributing classes i.e 5th, 6th^,^ and 7th having frequencies 44, 22, and 87 respectively were further divided into 5, 3, 11 sub-intervals, fuzzy sets $X_{i}$, i=1,2,3,…,25 were applied in the fuzzification process. By using eq. (3), we can fit our proposed model as in eq. (4) to defuzzified data as under:

**Table 1:** Growth rate against price (USD/Barrel) in WTi market.

Date	Price ($/BBL)	Growth Rate (T)	Date	Price ($/BBL)	Growth Rate (T)	Date	Price ($/BBL)	Growth Rate (T)	Date	Price ($/BBL)	Growth Rate (T)
2/17/2022	91.76	–	–	–	–	7/7/2022	102.73	0.042627	9/16/2022	85.11	0.000118
2/18/2022	91.07	−0.00752	5/2/2022	105.17	0.004585	7/8/2022	104.79	0.020053	9/19/2022	85.73	0.007285
2/20/2022	91.62	0.006039	5/3/2022	102.41	−0.02624	7/11/2022	104.09	−0.00668	9/20/2022	84.45	−0.01493
2/21/2022	92.65	0.011242	5/4/2022	107.81	0.052729	7/12/2022	95.84	−0.07926	9/21/2022	82.94	−0.01788
2/22/2022	92.35	−0.00324	5/5/2022	108.26	0.004174	7/13/2022	96.3	0.0048	9/22/2022	83.49	0.006631
2/23/2022	92.1	−0.00271	June 5, 2022	109.77	0.013948	7/14/2022	95.78	−0.0054	9/23/2022	78.74	−0.05689
2/24/2022	92.81	0.007709	September 5, 2022	103.09	−0.06085	7/15/2022	97.59	0.018897	9/26/2022	76.71	−0.02578
2/25/2022	91.59	−0.01315	October 5, 2022	99.76	−0.0323	7/18/2022	102.6	0.051337	9/27/2022	78.5	0.023335
2/28/2022	95.72	0.045092	November 5, 2022	105.71	0.059643	7/19/2022	104.22	0.015789	9/28/2022	82.15	0.046497
January 3, 2022	103.41	0.080338	December 5, 2022	106.13	0.003973	7/20/2022	102.26	−0.01881	9/29/2022	81.23	−0.0112
February 3, 2022	110.6	0.069529	5/13/2022	110.49	0.041082	7/21/2022	96.35	−0.05779	9/30/2022	79.49	−0.02142
March 3, 2022	107.67	−0.02649	5/16/2022	114.2	0.033578	7/22/2022	94.7	−0.01713	March 10, 2022	83.63	0.052082
April 3, 2022	115.68	0.074394	5/17/2022	112.4	−0.01576	7/25/2022	96.7	0.021119	April 10, 2022	86.52	0.034557
July 3, 2022	119.4	0.032158	5/18/2022	109.59	−0.025	7/26/2022	94.98	−0.01779	May 10, 2022	87.76	0.014332
August 3, 2022	123.7	0.036013	5/19/2022	112.21	0.023907	7/27/2022	97.26	0.024005	June 10, 2022	88.45	0.007862
September 3, 2022	108.7	−0.12126	5/20/2022	113.23	0.00909	7/28/2022	96.42	−0.00864	July 10, 2022	92.64	0.047371
October 3, 2022	106.02	−0.02466	5/23/2022	110.29	−0.02596	7/29/2022	98.62	0.022817	October 10, 2022	91.13	−0.0163
November 3, 2022	109.33	0.031221	5/24/2022	109.77	−0.00471	January 8, 2022	93.89	−0.04796	November 10, 2022	89.35	−0.01953
3/14/2022	103.01	−0.05781	5/25/2022	110.33	0.005102	February 8, 2022	94.42	0.005645	December 10, 2022	87.27	−0.02328
3/15/2022	96.44	−0.06378	5/26/2022	114.09	0.03408	March 8, 2022	90.66	−0.03982	10/13/2022	89.11	0.021084
3/16/2022	95.04	−0.01452	5/27/2022	115.07	0.00859	April 8, 2022	88.54	−0.02338	10/14/2022	85.61	−0.03928
3/17/2022	102.98	0.083544	5/29/2022	115.61	0.004693	May 8, 2022	89.01	0.005308	10/17/2022	85.46	−0.00175
3/18/2022	104.7	0.016702	5/30/2022	117.61	0.0173	August 8, 2022	90.76	0.019661	10/18/2022	82.82	−0.03089
3/21/2022	112.12	0.070869	5/31/2022	114.67	−0.025	September 8, 2022	90.5	−0.00286	10/19/2022	85.55	0.032963
3/22/2022	111.76	−0.00321	6/1/2022	115.26	0.005145	8/10/2022	91.93	0.015801	10/20/2022	85.98	0.005026
3/23/2022	114.93	0.028364	6/2/2022	116.87	0.013968	8/11/2022	94.34	0.026216	10/21/2022	85.05	−0.01082
3/24/2022	112.34	−0.02254	6/3/2022	118.87	0.017113	8/12/2022	92.09	−0.02385	10/24/2022	84.58	−0.00553
3/25/2022	113.9	0.013886	6/6/2022	118.5	−0.00311	8/15/2022	89.41	−0.0291	10/25/2022	85.32	0.008749
3/28/2022	105.96	−0.06971	6/7/2022	119.41	0.007679	8/16/2022	86.53	−0.03221	10/26/2022	87.91	0.030356
3/29/2022	104.24	−0.01623	6/8/2022	122.11	0.022611	8/17/2022	88.11	0.01826	10/27/2022	89.08	0.013309
3/30/2022	107.82	0.034344	6/9/2022	121.51	−0.00491	8/18/2022	90.5	0.027125	10/28/2022	87.9	−0.01325
3/31/2022	100.28	−0.06993	6/10/2022	120.67	−0.00691	8/19/2022	90.77	0.002983	10/31/2022	86.53	−0.01559
4/1/2022	99.27	−0.01007	6/13/2022	120.93	0.002155	8/22/2022	90.23	−0.00595	11/1/2022	88.37	0.021264
4/4/2022	103.28	0.040395	6/14/2022	118.93	−0.01654	8/23/2022	93.74	0.038901	11/2/2022	90	0.018445
4/5/2022	101.96	−0.01278	6/15/2022	115.31	−0.03044	8/24/2022	94.89	0.012268	11/3/2022	88.17	−0.02033
4/6/2022	96.23	−0.0562	6/16/2022	117.59	0.019773	8/25/2022	92.52	−0.02498	11/4/2022	92.61	0.050357
4/7/2022	96.03	−0.00208	6/17/2022	109.56	−0.06829	8/26/2022	93.06	0.005837	11/7/2022	91.79	−0.00885
4/8/2022	98.26	0.023222	6/19/2022	108.94	−0.00566	8/29/2022	97.01	0.042446	11/8/2022	88.91	−0.03138
4/11/2022	94.29	−0.0404	6/20/2022	108.84	−0.00092	8/30/2022	91.64	−0.05536	11/9/2022	85.83	−0.03464
4/12/2022	100.6	0.066921	6/21/2022	110.65	0.01663	8/31/2022	89.55	−0.02281	11/10/2022	86.47	0.007457
4/13/2022	104.25	0.036282	6/22/2022	106.19	−0.04031	9/1/2022	86.61	−0.03283	11/11/2022	88.96	0.028796
4/14/2022	106.95	0.025899	6/23/2022	104.27	−0.01808	9/2/2022	86.87	0.003002	11/14/2022	85.87	−0.03473
4/18/2022	108.21	0.011781	6/24/2022	107.62	0.032128	9/4/2022	88.25	0.015886	11/15/2022	86.92	0.012228
4/19/2022	102.56	−0.05221	6/27/2022	109.57	0.018119	9/5/2022	88.96	0.008045	11/16/2022	85.59	−0.0153
4/20/2022	102.75	0.001853	6/28/2022	111.76	0.019987	9/6/2022	86.88	−0.02338	11/17/2022	81.64	−0.04615
4/21/2022	103.79	0.010122	6/29/2022	109.78	−0.01772	9/7/2022	81.94	−0.05686	11/18/2022	80.08	−0.01911
4/22/2022	102.07	−0.01657	6/30/2022	105.76	−0.03662	9/8/2022	83.54	0.019526	11/20/2022	80.2	0.001499
4/25/2022	98.54	−0.03458	7/1/2022	108.43	0.025246	9/9/2022	86.79	0.038904	11/21/2022	80.36	0.001995
4/26/2022	101.7	0.032068	7/3/2022	108.19	−0.00221	9/12/2022	87.78	0.011407	11/22/2022	81.31	0.011822
4/27/2022	102.02	0.003147	7/4/2022	110.4	0.020427	9/13/2022	87.31	−0.00535	11/23/2022	81.08	−0.00283
4/28/2022	105.36	0.032739	7/5/2022	99.5	−0.09873	9/14/2022	88.48	0.013401			
4/29/2022	104.69	−0.00636	7/6/2022	98.53	−0.00975	9/15/2022	85.1	−0.0382

**Fig. 3:**
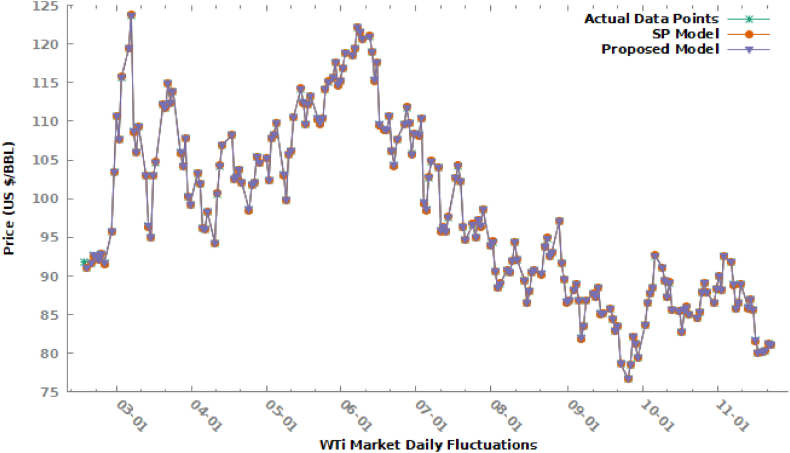
Comparison of proposed model to SP model against observed data.

In Table 2, the proposed model fitted against observed values using defuzzified values and was compared graphically to Stevenson and Porter's (SP) model [14] in Fig. 3.

**Table 2:** Fitting proposed model (Y_t_) against actual crude oil price (USD/BBL).

Date	Price	T	Fuzzy Sets	t	Proposed Model	Date	Price	T	Fuzzy Sets	t	Proposed Model
2/17/2022	91.76	–	–	–	–	7/7/2022	102.73	0.0426	X24	0.029739	102.7303
2/18/2022	91.07	−0.0075	X3	−0.06008	91.0694	7/8/2022	104.79	0.0201	X15	0.063781	104.7906
2/20/2022	91.62	0.0060	X9	−0.01718	91.61983	7/11/2022	104.09	−0.0067	X9	−0.01718	104.0898
2/21/2022	92.65	0.0112	X6	−0.01192	92.64988	7/12/2022	95.84	−0.0793	X2	−0.08405	95.83916
2/22/2022	92.35	−0.0032	X4	−0.04138	92.34959	7/13/2022	96.3	0.0048	X12	−0.01192	96.29988
2/23/2022	92.1	−0.0027	X6	−0.03024	92.0997	7/14/2022	95.78	−0.0054	X8	−0.03024	95.7797
2/24/2022	92.81	0.0077	X12	−0.01192	92.80988	7/15/2022	97.59	0.0189	X13	0.063781	97.59064
2/25/2022	91.59	−0.0131	X7	−0.03024	91.5897	7/18/2022	102.6	0.0513	X24	0.029739	102.6003
2/28/2022	95.72	0.0451	X24	0.029739	95.7203	7/19/2022	104.22	0.0158	X13	0.063781	104.2206
January 3, 2022	103.41	0.0803	X25	0.05068	103.4105	7/20/2022	102.26	−0.0188	X8	−0.03024	102.2597
February 3, 2022	110.6	0.0695	X25	0.05068	110.6005	7/21/2022	96.35	−0.0578	X2	−0.08405	96.34916
March 3, 2022	107.67	−0.0265	X6	−0.03024	107.6697	7/22/2022	94.7	−0.0171	X7	−0.03024	94.6997
April 3, 2022	115.68	0.0744	X25	0.05068	115.6805	7/25/2022	96.7	0.0211	X17	0.015275	96.70015
July 3, 2022	119.4	0.0322	X21	0.015275	119.4002	7/26/2022	94.98	−0.0178	X6	−0.03024	94.9797
August 3, 2022	123.7	0.0360	X23	0.017962	123.7002	7/27/2022	97.26	0.0240	X17	0.015275	97.26015
September 3, 2022	108.7	−0.1213	X1	−0.10108	108.699	7/28/2022	96.42	−0.0086	X8	−0.03024	96.4197
October 3, 2022	106.02	−0.0247	X6	−0.03024	106.0197	7/29/2022	98.62	0.0228	X17	0.015275	98.62015
November 3, 2022	109.33	0.0312	X21	0.015275	109.3302	January 8, 2022	93.89	−0.0480	X4	−0.04138	93.88959
3/14/2022	103.01	−0.0578	X3	−0.06008	103.0094	February 8, 2022	94.42	0.0056	X13	0.063781	94.42064
3/15/2022	96.44	−0.0638	X3	−0.06008	96.4394	March 8, 2022	90.66	−0.0398	X4	−0.04138	90.65959
3/16/2022	95.04	−0.0145	X4	−0.04138	95.03959	April 8, 2022	88.54	−0.0234	X6	−0.03024	88.5397
3/17/2022	102.98	0.0835	X25	0.05068	102.9805	May 8, 2022	89.01	0.0053	X13	0.063781	89.01064
3/18/2022	104.7	0.0167	X13	0.063781	104.7006	August 8, 2022	90.76	0.0197	X12	−0.01192	90.75988
3/21/2022	112.12	0.0709	X25	0.05068	112.1205	September 8, 2022	90.5	−0.0029	X7	−0.03024	90.4997
3/22/2022	111.76	−0.0032	X5	−0.03246	111.7597	8/10/2022	91.93	0.0158	X13	0.063781	91.93064
3/23/2022	114.93	0.0284	X19	0.015275	114.9302	8/11/2022	94.34	0.0262	X17	0.015275	94.34015
3/24/2022	112.34	−0.0225	X6	−0.03024	112.3397	8/12/2022	92.09	−0.0238	X5	−0.03246	92.08968
3/25/2022	113.9	0.0139	X12	−0.01192	113.8999	8/15/2022	89.41	−0.0291	X4	−0.04138	89.40959
3/28/2022	105.96	−0.0697	X3	−0.06008	105.9594	8/16/2022	86.53	−0.0322	X3	−0.06008	86.5294
3/29/2022	104.24	−0.0162	X8	−0.03024	104.2397	8/17/2022	88.11	0.0183	X12	−0.01192	88.10988
3/30/2022	107.82	0.0343	X22	0.015275	107.8202	8/18/2022	90.5	0.0271	X16	0.015275	90.50015
3/31/2022	100.28	−0.0699	X3	−0.06008	100.2794	8/19/2022	90.77	0.0030	X11	−0.00748	90.76993
4/1/2022	99.27	−0.0101	X9	−0.01718	99.26983	8/22/2022	90.23	−0.0059	X8	−0.03024	90.2297
4/4/2022	103.28	0.0404	X24	0.029739	103.2803	8/23/2022	93.74	0.0389	X20	0.015275	93.74015
4/5/2022	101.96	−0.0128	X8	−0.03024	101.9597	8/24/2022	94.89	0.0123	X18	0.015275	94.89015
4/6/2022	96.23	−0.0562	X3	−0.06008	96.2294	8/25/2022	92.52	−0.0250	X6	−0.03024	92.5197
4/7/2022	96.03	−0.0021	X5	−0.03246	96.02968	8/26/2022	93.06	0.0058	X11	−0.00748	93.05993
4/8/2022	98.26	0.0232	X16	0.015275	98.26015	8/29/2022	97.01	0.0424	X23	0.017962	97.01018
4/11/2022	94.29	−0.0404	X4	−0.04138	94.28959	8/30/2022	91.64	−0.0554	X3	−0.06008	91.6394
4/12/2022	100.6	0.0669	X25	0.05068	100.6005	8/31/2022	89.55	−0.0228	X9	−0.01718	89.54983
4/13/2022	104.25	0.0363	X23	0.017962	104.2502	9/1/2022	86.61	−0.0328	X7	−0.03024	86.6097
4/14/2022	106.95	0.0259	X18	0.015275	106.9502	9/2/2022	86.87	0.0030	X10	−0.00921	86.86991
4/18/2022	108.21	0.0118	X12	−0.01192	108.2099	9/4/2022	88.25	0.0159	X12	−0.01192	88.24988
4/19/2022	102.56	−0.0522	X5	−0.03246	102.5597	9/5/2022	88.96	0.0080	X11	−0.00748	88.95993
4/20/2022	102.75	0.0019	X13	0.063781	102.7506	9/6/2022	86.88	−0.0234	X7	−0.03024	86.8797
4/21/2022	103.79	0.0101	X12	−0.01192	103.7899	9/7/2022	81.94	−0.0569	X3	−0.06008	81.9394
4/22/2022	102.07	−0.0166	X7	−0.03024	102.0697	9/8/2022	83.54	0.0195	X14	−0.01192	83.53988
4/25/2022	98.54	−0.0346	X4	−0.04138	98.53959	9/9/2022	86.79	0.0389	X19	0.015275	86.79015
4/26/2022	101.7	0.0321	X21	0.015275	101.7002	9/12/2022	87.78	0.0114	X12	−0.01192	87.77988
4/27/2022	102.02	0.0031	X12	−0.01192	102.0199	9/13/2022	87.31	−0.0054	X8	−0.03024	87.3097
4/28/2022	105.36	0.0327	X21	0.015275	105.3602	9/14/2022	88.48	0.0134	X14	−0.01192	88.47988
4/29/2022	104.69	−0.0064	X10	−0.00921	104.6899	9/15/2022	85.1	−0.0382	X6	−0.03024	85.0997
5/2/2022	105.17	0.0046	X12	−0.01192	105.1699	9/16/2022	85.11	0.0001	X10	−0.00921	85.10991
5/3/2022	102.41	−0.0262	X5	−0.03246	102.4097	9/19/2022	85.73	0.0073	X11	−0.00748	85.72993
5/4/2022	107.81	0.0527	X24	0.029739	107.8103	9/20/2022	84.45	−0.0149	X5	−0.03246	84.44968
5/5/2022	108.26	0.0042	X24	0.029739	108.2603	9/21/2022	82.94	−0.0179	X6	−0.03024	82.9397
June 5, 2022	109.77	0.0139	X12	−0.01192	109.7699	9/22/2022	83.49	0.0066	X12	−0.01192	83.48988
September 5, 2022	103.09	−0.0609	X3	−0.06008	103.0894	9/23/2022	78.74	−0.0569	X3	−0.06008	78.7394
October 5, 2022	99.76	−0.0323	X5	−0.03246	99.75968	9/26/2022	76.71	−0.0258	X4	−0.04138	76.70959
November 5, 2022	105.71	0.0596	X24	0.029739	105.7103	9/27/2022	78.5	0.0233	X16	0.015275	78.50015
December 5, 2022	106.13	0.0040	X23	0.017962	106.1302	9/28/2022	82.15	0.0465	X24	0.029739	82.1503
5/13/2022	110.49	0.0411	X23	0.017962	110.4902	9/29/2022	81.23	−0.0112	X6	−0.03024	81.2297
5/16/2022	114.2	0.0336	X22	0.015275	114.2002	9/30/2022	79.49	−0.0214	X3	−0.06008	79.4894
5/17/2022	112.4	−0.0158	X8	−0.03024	112.3997	March 10, 2022	83.63	0.0521	X25	0.05068	83.63051
5/18/2022	109.59	−0.0250	X6	−0.03024	109.5897	April 10, 2022	86.52	0.0346	X22	0.015275	86.52015
5/19/2022	112.21	0.0239	X16	0.015275	112.2102	May 10, 2022	87.76	0.0143	X14	−0.01192	87.75988
5/20/2022	113.23	0.0091	X13	0.063781	113.2306	June 10, 2022	88.45	0.0079	X12	−0.01192	88.44988
5/23/2022	110.29	−0.0260	X6	−0.03024	110.2897	July 10, 2022	92.64	0.0474	X23	0.017962	92.64018
5/24/2022	109.77	−0.0047	X2	−0.08405	109.7692	October 10, 2022	91.13	−0.0163	X8	−0.03024	91.1297
5/25/2022	110.33	0.0051	X11	−0.00748	110.3299	November 10, 2022	89.35	−0.0195	X7	−0.03024	89.3497
5/26/2022	114.09	0.0341	X22	0.015275	114.0902	December 10, 2022	87.27	−0.0233	X6	−0.03024	87.2697
5/27/2022	115.07	0.0086	X15	0.063781	115.0706	10/13/2022	89.11	0.0211	X15	0.063781	89.11064
5/29/2022	115.61	0.0047	X14	−0.01192	115.6099	10/14/2022	85.61	−0.0393	X5	−0.03246	85.60968
5/30/2022	117.61	0.0173	X12	−0.01192	117.6099	10/17/2022	85.46	−0.0018	X9	−0.01718	85.45983
5/31/2022	114.67	−0.0250	X6	−0.03024	114.6697	10/18/2022	82.82	−0.0309	X7	−0.03024	82.8197
6/1/2022	115.26	0.0051	X12	−0.01192	115.2599	10/19/2022	85.55	0.0330	X17	0.015275	85.55015
6/2/2022	116.87	0.0140	X17	0.015275	116.8702	10/20/2022	85.98	0.0050	X13	0.063781	85.98064
6/3/2022	118.87	0.0171	X18	0.015275	118.8702	10/21/2022	85.05	−0.0108	X8	−0.03024	85.0497
6/6/2022	118.5	−0.0031	X6	−0.03024	118.4997	10/24/2022	84.58	−0.0055	X4	−0.04138	84.57959
6/7/2022	119.41	0.0077	X12	−0.01192	119.4099	10/25/2022	85.32	0.0087	X12	−0.01192	85.31988
6/8/2022	122.11	0.0226	X22	0.015275	122.1102	10/26/2022	87.91	0.0304	X19	0.015275	87.91015
6/9/2022	121.51	−0.0049	X8	−0.03024	121.5097	10/27/2022	89.08	0.0133	X12	−0.01192	89.07988
6/10/2022	120.67	−0.0069	X9	−0.01718	120.6698	10/28/2022	87.9	−0.0132	X7	−0.03024	87.8997
6/13/2022	120.93	0.0022	X13	0.063781	120.9306	10/31/2022	86.53	−0.0156	X5	−0.03246	86.52968
6/14/2022	118.93	−0.0165	X7	−0.03024	118.9297	11/1/2022	88.37	0.0213	X16	0.015275	88.37015
6/15/2022	115.31	−0.0304	X2	−0.08405	115.3092	11/2/2022	90	0.0184	X11	−0.00748	89.99993
6/16/2022	117.59	0.0198	X13	0.063781	117.5906	11/3/2022	88.17	−0.0203	X6	−0.03024	88.1697
6/17/2022	109.56	−0.0683	X1	−0.10108	109.559	11/4/2022	92.61	0.0504	X24	0.029739	92.6103
6/19/2022	108.94	−0.0057	X9	−0.01718	108.9398	11/7/2022	91.79	−0.0089	X7	−0.03024	91.7897
6/20/2022	108.84	−0.0009	X10	−0.00921	108.8399	11/8/2022	88.91	−0.0314	X3	−0.06008	88.9094
6/21/2022	110.65	0.0166	X13	0.063781	110.6506	11/9/2022	85.83	−0.0346	X2	−0.08405	85.82916
6/22/2022	106.19	−0.0403	X4	−0.04138	106.1896	11/10/2022	86.47	0.0075	X12	−0.01192	86.46988
6/23/2022	104.27	−0.0181	X7	−0.03024	104.2697	11/11/2022	88.96	0.0288	X17	0.015275	88.96015
6/24/2022	107.62	0.0321	X22	0.015275	107.6202	11/14/2022	85.87	−0.0347	X4	−0.04138	85.86959
6/27/2022	109.57	0.0181	X16	0.015275	109.5702	11/15/2022	86.92	0.0122	X13	0.063781	86.92064
6/28/2022	111.76	0.0200	X15	0.063781	111.7606	11/16/2022	85.59	−0.0153	X6	−0.03024	85.5897
6/29/2022	109.78	−0.0177	X7	−0.03024	109.7797	11/17/2022	81.64	−0.0462	X2	−0.08405	81.63916
6/30/2022	105.76	−0.0366	X4	−0.04138	105.7596	11/18/2022	80.08	−0.0191	X8	−0.03024	80.0797
7/1/2022	108.43	0.0252	X22	0.015275	108.4302	11/20/2022	80.2	0.0015	X13	0.063781	80.20064
7/3/2022	108.19	−0.0022	X8	−0.03024	108.1897	11/21/2022	80.36	0.0020	X11	−0.00748	80.35993
7/4/2022	110.4	0.0204	X16	0.015275	110.4002	11/22/2022	81.31	0.0118	X12	−0.01192	81.30988
7/5/2022	99.5	−0.0987	X1	−0.10108	99.49899	11/23/2022	81.08	−0.0028	X8	−0.03024	81.0797

In Fig. 3, we see that the proposed model, the plot overlaps the SP model and as a result, shows a single curve, that fits the observed data well than the SP model. It is a clear indication of better forecast accuracy than the SP Model. However, the forecast accuracy may be increased if the parameter under (3) is estimated rather than using specified values as 0.5 each. The parameters $\gamma_{1}$, $\gamma_{2}$, $\delta_{1}$ and $\delta_{2}$ are to be optimized for said purpose. The general form of adjusted defuzzified $t_{j}^{adj}$ is given as under in eq. (5):

(5) $$t_{j}^{adj}=\left\{ \begin{array}{cc} \frac{1+\gamma_{1}}{\frac{1}{a_{1}}+\frac{\gamma_{1}}{a_{2}}} & j=1\\ \frac{\delta_{1}+1+\delta_{2}}{\frac{\delta_{1}}{a_{j-1}}+\frac{1}{a_{j}}+\frac{\delta_{2}}{a_{j+1}}} & 2\leq j\leq m-1\\ \frac{\gamma_{2}+1}{\frac{\gamma_{2}}{a_{j-1}}+\frac{1}{a_{j}}} & j=m \end{array}\right.$$

**Fig. 4:**
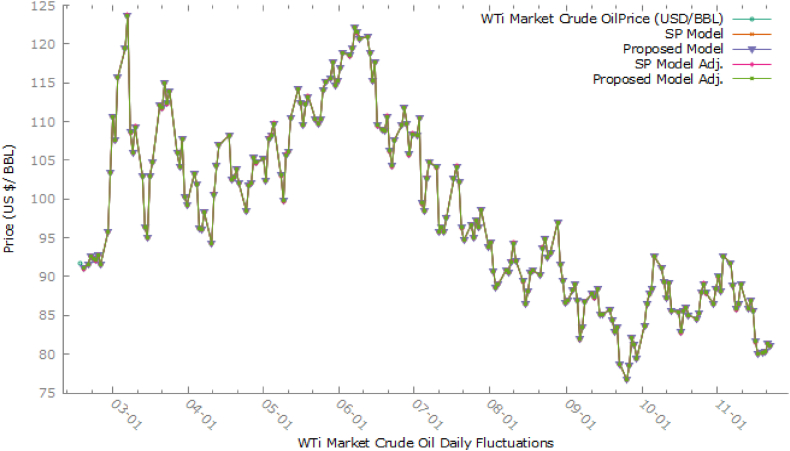
Comparison of methods with specified and optimized parameters.

For optimization of these parameters, a simultaneous equation system through Maple 17 was used and got the optimized values as $\gamma_{1}=$ 0.5000000029, $\gamma_{2}=$ 0.499999996, $\delta_{1}$ = 12.00840071 and $\delta_{2}$ = 53.79319947. We observed a huge difference in the optimized values of the last two parameters as compared to specified values. Fig. 4 shows the comparison of all studied methods in this article.

**Fig. 5:**
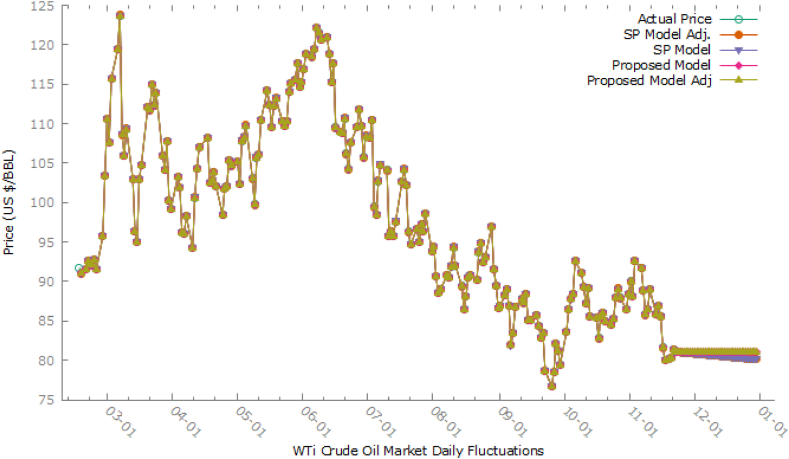
Comparison in models forecasting.

As it is shown in Fig. 4, the plot of the proposed model with optimized parameters overlaps all other model plots and is more visible than all methods in this scenario. So, it is evident that the proposed model improved the forecasting accuracy by having optimized parameters. So, models with specific parametric values have less accuracy than optimized ones. The model forecasting performance is given as a Fig. 5.

In Fig. 5, we tried to check the forecasting performance of models i.e. from Nov 24, 2022, to Dec 31, 2022. As like Fig. 4, the plot of the proposed model with optimized parameters overlaps all other model plots and is more visible than all methods in this scenario. So, it is evident that the proposed model with optimized values is performing better model forecasting than all others. So we may conclude that in fuzzy time series, usually optimized parameters play important in improving the forecasting accuracy of the model. It shows that up to Dec 31, 2022, the price of crude oil ($/barrel) will keep lowering at a very slow rate during the Ukraine-Russia conflict and is good for the worldwide economy, if the tendency continues to fall for a long.

**ARIMA (1,0,0)** was utilized in the analysis as conventional time series through auto.arima function in Gretl, the significance of parameters was chosen by AIC and BIC criteria. The result of model forecasting with a complete summary is given in Table 3, Table 4.

**Table 3:** ARIMA(1,0,0) model estimation.

Parameters	Coefficient	Std. Error	z	p-value
Constant	95.9559	5.10247	18.81	6.77e-079 ***
$\emptyset_{1}$	0.959289	0.0189186	50.71	0.0000 ***

**Table 4:** ARIMA(1,0,0) model summary.

Mean dependent var	98.14790	S.D. dependent var	11.39230
Mean of innovations	0.034315	S.D. of innovations	3.250831
R-Square	0.918197	Adjusted R-Square	0.918197
Log-likelihood	−533.8234	Akaike criterion	1073.647
Schwarz criterion	1083.616	Hannan-Quinn	1077.679

**Fig. 6:**
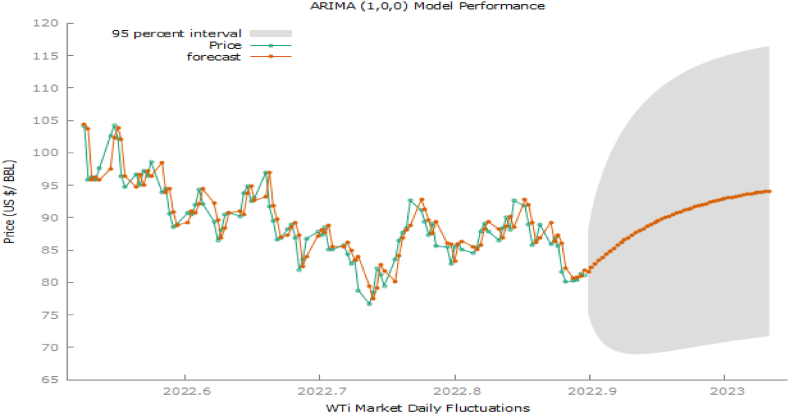
Arima (1,0,0) forecasting to actual data with 95 % C.I.

From the above ARIMA (1,0,0) model, the autoregressive term is highly significant. Fig. 6 gives us a vibrant look into in-sample and out-of-sample forecasting with real-time forecasting with a 95 % confidence interval.

## 4 Results

The overall comparison summary of all models is given in Table 5, showing the MAPE of all models i.e. Proposed, SP, ARIMA, Artificial Neural Networks (ANN) [15], Support Vector Machine (SVM) [16] and XGBoost [17] models with respective relative efficiency, keeping the SP model as the base model with a relative efficiency of 100 %, it is clear from the table that the Proposed Model has higher relative efficiency (marked as bold) than all competitive methods in this study. Table 5 shows that the proposed model with adjusted parameters has greater forecasting accuracy than all competitive methods.

**Table 5:** Overall comparison of all models.

Model		MAPE	Forecast	95 % C.I	Relative Efficiency
Specified Parameters	SP Model	0.06008046	82.1138	73.9216–104.1928	100
	Proposed Model	**0.000659717**	81.8441	76.5761–102.2801	**9107.005**
Adjusted Parameters	SP Model	0.046197248	80.1294	72.8832–103.1239	130.052
	Proposed Model	**0.000507272**	78.8441	75.2191–98.2891	**11843.83526**
ARIMA (1,0,0)		2.4946	95.72	83.1712–118.2891	2.41
ANN		0.0782218	84.1397	74.2118–107.9123	76.8078
SVM		0.0779431	86.4309	75.2391–108.5921	77.0825
XGBoost		0.0472914	80.6723	71.5364–101.8732	127.0431

## 5 Discussion

In this article we tried to find out the best forecasting method for the crude oil price (USD/Barrel) WTi international market using the dataset from February 17, 2022, to Nov 23, 2022, in assessing the international market against Ukraine-Russia War which was started on February 24, 2022, and going on. We found that our proposed model is the best-performing forecasting model versus other fuzzy time series, ARIMA, ANN, SVM and XGBoost models. The in-sample and the out-sample forecast show Proposed Model has the least MAPE against the base model. The base SP model [14], which was outperformed by proposed model in both fitting and forecasting to real-time data in having specified parameters as well as with estimated optimized parameters. We further made its comparison to ARIMA(1,0,0), ANN, SVM and XGBoost models for better insight performance of the proposed model and found it better than it. Overall, we conclude that FTS models perform better than conventional time series models. The main issue in the FTS model in forming the universe of discourse and its partitioning. MAPE is highly dependent on it. MAPE will be least if the partitioning is least otherwise not.

Our finding, out of the sample forecast shows that the international market WTi crude oil prices (US $/BBL) will keep lowering with every passing day after hitting the record highest price in history. The lowering price effect may be seen in the world economy as initial war days were not so good for it is higher prices. Higher prices of crude oil have huge consequences on oil-importing countries and their GDP is directly linked to US Dollar. So, Ukraine- Russia war bears huge economic consequences for the whole world.

## 6 Conclusion

The key findings of the article are given below.

●The proposed method in fuzzy time series is performing better than its competitors for both specified and optimized parameters.
●There is no unique universe of discourse as in SP model claimed, i.e. in the previous literature. The choice of UoD is a subjective approach.
●Forecasting through interval estimation gives more information than point forecasting.
●Relative efficiency is a good addition in research for checking model accuracy.

## 7 Future perspective

The further research directions on same study are as under.

●Assessment of global economy in the presence of Ukraine-Russia War using Multivariate Fuzzy Time Series.
●Machine Learning Techniques may also be utilized for deep insight knowledge.
●Comparative analysis of multivariate time series models versus fuzzy and machine learning methods can also be adopted.

## Data availability statement

The data was taken from WTi website. i.e. https://www.investing.com/commodities/crude-oil.

## Additional information

No additional information is available for this paper.

## Funding statement

No funding is available for carrying out this research work.

## CRediT authorship contribution statement

**Muhammad Bilal:** Writing – review & editing, Writing – original draft, Methodology, Conceptualization. **Muhammad Aamir:** Supervision, Software, Project administration, Investigation, Formal analysis. **Saleem Abdullah:** Validation, Resources, Project administration, Conceptualization. **Faisal Khan:** Funding acquisition, Formal analysis.

## Declaration of competing interest

The authors declare that they have no known competing financial interests or personal relationships that could have appeared to influence the work reported in this paper.
